# Cell Metabolism Control Through O-GlcNAcylation of STAT5: A Full or Empty Fuel Tank Makes a Big Difference for Cancer Cell Growth and Survival

**DOI:** 10.3390/ijms20051028

**Published:** 2019-02-27

**Authors:** Manuel Rauth, Patricia Freund, Anna Orlova, Stefan Grünert, Nikola Tasic, Xiaonan Han, Hai-Bin Ruan, Heidi A. Neubauer, Richard Moriggl

**Affiliations:** 1 Institute of Animal Breeding and Genetics, University of Veterinary Medicine Vienna, 1210 Vienna, Austria; Manuel.Rauth@vetmeduni.ac.at (M.R.); Patricia.Freund@vetmeduni.ac.at (P.F.); Anna.Orlova@lbicr.lbg.ac.at (A.O.); Heidi.Neubauer@vetmeduni.ac.at (H.A.N.); 2 Ludwig Boltzmann Institute for Cancer Research, 1090 Vienna, Austria; 3 Biolution GmbH, 1030 Vienna, Austria; stefan.gruenert@biolution.net (S.G.); tasic@biolution.net (N.T.); 4 Key Laboratory of Human Disease Comparative Medicine, the Ministry of Health, Institute of Laboratory Animal Sciences (ILAS), Beijing 100730, China; Xiaonan.Han@cchmc.org; 5 Chinese Academy of Medical Science (CAMS) and Peking Union Medical College (PUMC), Beijing 100006, China; 6 Division of Gastroenterology, Hepatology and Nutrition, Cincinnati Children’s Hospital Medical Center (CCHMC), Cincinnati, OH 45229-3026, USA; 7 Department of Integrative Biology and Physiology, University of Minnesota Medical School, Minneapolis, MN 55455, USA; hruan@umn.edu; 8 Medical University Vienna, Vienna 1090, Austria

**Keywords:** STAT5A, STAT5B, STAT3, JAK kinases, tyrosine phosphorylation, O-GlcNAcylation, O-GlcNAc transferase, O-GlcNAcase

## Abstract

O-GlcNAcylation is a post-translational modification that influences tyrosine phosphorylation in healthy and malignant cells. O-GlcNAc is a product of the hexosamine biosynthetic pathway, a side pathway of glucose metabolism. It is essential for cell survival and proper gene regulation, mirroring the metabolic status of a cell. STAT3 and STAT5 proteins are essential transcription factors that can act in a mutational context-dependent manner as oncogenes or tumor suppressors. They regulate gene expression for vital processes such as cell differentiation, survival, or growth, and are also critically involved in metabolic control. The role of STAT3/5 proteins in metabolic processes is partly independent of their transcriptional regulatory role, but is still poorly understood. Interestingly, STAT3 and STAT5 are modified by O-GlcNAc in response to the metabolic status of the cell. Here, we discuss and summarize evidence of O-GlcNAcylation-regulating STAT function, focusing in particular on hyperactive STAT5A transplant studies in the hematopoietic system. We emphasize that a single O-GlcNAc modification is essential to promote development of neoplastic cell growth through enhancing STAT5A tyrosine phosphorylation. Inhibition of O-GlcNAcylation of STAT5A on threonine 92 lowers tyrosine phosphorylation of oncogenic STAT5A and ablates malignant transformation. We conclude on strategies for new therapeutic options to block O-GlcNAcylation in combination with tyrosine kinase inhibitors to target neoplastic cancer cell growth and survival.

## 1. Introduction

The metabolic state of tumor cells dramatically differs from the metabolism of healthy cells, and this largely defines the proliferation and expansion of malignancies. In the 1920s, the German biochemist and Nobel Prize laureate Otto H. Warburg observed the phenomenon of tumor cells radically changing their metabolism, which was termed the “Warburg effect”. It was demonstrated that cancer cells downregulate mitochondrial function and increase the production of lactate through cytoplasmic enzyme cascades. Lactate can enter the tricarboxylic acid (TCA) cycle to feed glutamate production, facilitating lipid synthesis or neoangiogenesis in a hypoxic environment [1]. Since this discovery, many researchers have focused on understanding the advantages that cancer cells obtain by undergoing a more expensive and less efficient method of energy production. For a long time, the role of anaerobic glycolysis in cancer was neglected. However, recent cancer and immunology research has provided a better understanding of the consequences and advantages that changes in metabolic processes bring for normal or neoplastic cells [2,3,4]. Importantly, cancer cells are highly dependent on glucose and glutamine with respect to energy production. As the uptake of these compounds is crucial, the intracellular concentration of these molecules increases. This leads to increased glycolysis in cancer cells and thereby an increased flux through the hexosamine biosynthetic pathway (HBP), which, in the end, provides the cell with uridine diphosphate-acetylglucosamine (UDP-GlcNAc). This further serves as a donor for O-linked β-N-acetylglucosamine (O-GlcNAc) modification of proteins [5]. Interestingly, increased O-GlcNAcylation of proteins necessary for the TCA cycle, such as pyruvate dehydrogenase protein X component (PDHX), disturbs their function and leads to a disruption of mitochondrial function [6]. Therefore, increased O-GlcNAcylation status in cancer cells may promote this switch from oxidative phosphorylation to the Warburg effect.

Our findings on the influence of O-GlcNAc modification on signal transducer and activator of transcription (STAT) protein function demonstrate that metabolic changes are indeed of fundamental importance for the development of aggressive cancer growth and survival. Experimental models were mainly based on insights of normal or oncogenic STAT5A signaling [7]. Here, we shed light on how the STAT3/5 transcription factors can integrate metabolic signals to contribute to oncogenic transformation, involving O-GlcNAcylation in association with excessive tyrosine phosphorylation.

## 2. Overview of the Janus Kinase Signal Transducer and Activator of Transcription (JAK-STAT) Signaling Pathway

**Figure 1:**
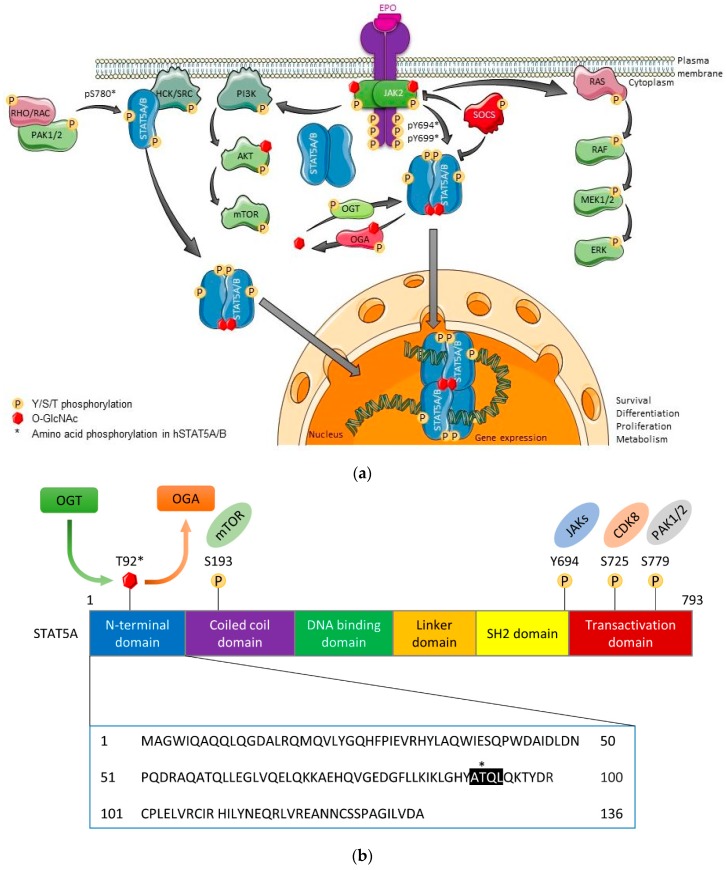
The JAK2-STAT5-SOCS pathway and domain structure of STAT5. (**a**) Upon cytokine binding to respective cytokine receptor chain(s), a conformational change in the transmembrane domain triggers activation of associated Janus kinase (JAK) kinases that bind to the BOX1 and BOX2 membrane proximal motifs. Subsequently, antiparallel STAT5 dimers are efficiently recruited and JAK dimers then activate STAT5 by tyrosine phosphorylation. STAT5 then undergoes a drastic conformational change to form parallel dimers. Furthermore, serine/threonine phosphorylation of STAT family members triggers shuttling in and out of the nucleus as well as transcriptional elongation. STATs are usually inactivated by tyrosine phosphatases, which are much more highly expressed than the rate limiting JAK kinases. In contrast, inhibition of cytokine receptor and JAK kinase signaling is executed via ubiquitination by SOCS family members, downstream targets of STATs that provide negative feedback control. JAK2 glycosylation is also observed (our unpublished data). (**b**) The STAT5A domain structure is schematically illustrated with the O-GlcNAc modification depicted at T92 (indicated by asterisk) within an ATQL motif (highlighted in black) in the N-domain. STATs consist of six domains: N-terminal domain (blue), coiled coil domain (purple), DNA binding domain (green), linker domain (orange), SH2 domain (yellow) and transcriptional activation/stability domain (red) [15,16,19]. Reported phosphorylation sites and respective kinases are also shown. OGT, O-GlcNAc transferase; OGA, O-GlcNAcase.

The Janus kinase (JAK)-STAT pathway is a core cancer pathway that transmits a signal from the cell surface to the nucleus through cooperation between JAK kinase and STAT transcription factors. It determines cellular fate, proliferation, and survival. However, it is also critically involved in the regulation of cell cycle arrest or in promoting senescence (Figure 1a). JAK-STAT signaling is initiated when two or more cytokine receptor chains interact upon stimulation by different ligand mediators, such as interleukins (IL), or upon binding of most myeloid or lymphoid cell-acting cytokines [8,9]. Cytokine receptors lack kinase domains, but upon ligand stimulation they can activate one or several members of the four JAK kinases, namely JAK1, JAK2, JAK3, and Tyrosine kinase (TYK) 2 [10,11].

Upon binding of a cytokine to its receptor complex, a slight conformational change occurs in the transmembrane domain of the cytokine receptor, allowing for a mechanical movement of the pseudokinase domain away from the kinase domain of the dimeric JAK complex. This promotes JAK autoactivation, triggering a phosphorylation cascade and subsequently the activation of a large number of key signaling molecules, including the STATs [12]. Overall, freeing of the JAK pseudokinase from the kinase domain leads to the subsequent formation of docking sites for downstream adaptor and effector proteins [13,14]. Growth factor receptors or G-protein coupled receptors were also shown to be able to activate STAT proteins upon ligand binding, either dependent or independent of JAK kinase activity. In cancer, gain-of-function (GOF) JAK mutations are frequent, resulting in constitutive JAK kinase activity that can further promote the activation of other core cancer pathways, such as RAS-RAF-MAPK or PI3K-AKT-mTOR (Figure 1a).

A vast number of cytokines, such as prolactin, erythropoietin (EPO), thrombopoietin (TPO), growth hormone, IL-2, IL-3, IL-4, IL-5, IL-7, IL-9, IL-15, IL-21, IL-31, oncostatin M (OSM) and granulocyte-macrophage colony-stimulating factor (GM-CSF) can activate STAT5. Upon tyrosine phosphorylation (pYSTAT), STAT proteins undergo a drastic conformational change to a transcriptionally active state by forming parallel homo- or heterodimers (e.g., STAT1/1, STAT1/3, STAT3/3, STAT5A/5A, STAT5B/5B, or STAT5A/5B) [15,16]. In the hematopoietic system, loss of STAT5 is associated with reduced blood cell lineage capacity, e.g., anemia or lymphopenia. In contrast, overexpression or hyperactivation of STAT5 is associated with leukemic growth or neoplastic transformation. For example, myeloproliferative neoplasms (MPN), chronic myeloid leukemia (CML), acute myeloid leukemia (AML), and various lymphomas are driven by increased pYSTAT5 levels [8,17,18].

The seven STAT family members (STAT1-4, STAT5A, STAT5B, and STAT6) control cell proliferation, survival, and metabolism, as well as regulate important cell fate decisions and tissue remodeling. The functions of STAT proteins are best described in hematopoiesis and immunity, where they play essential roles in the development of all hematopoietic lineages and, subsequently, proper functioning of immune cells controlling infectious disease, cancer, or autoimmune processes [20]. Outside of the blood, STAT proteins also possess important functions for sex determination, reproduction, body growth, neuro-endocrine processes, intestinal homeostasis, and injury repair, with specific and complex functions in the liver, kidney, ovary, colon, and epithelial glands (e.g., prostate, mammary or pituitary gland) [21,22,23,24].

STAT family members are between 750 and 850 amino acids long, and they consist of six conserved domains (Figure 1b) [7,8,18]. The N-terminal domain of STAT proteins is involved in receptor docking. Specifically, the N-domain of STAT1/3/4/5A/5B can participate in higher-order oligomer formation [25]. Furthermore, the N-domain of STAT1/3/5 is involved in interactions with nuclear hormone receptors, such as the stress hormone receptor/glucocorticoid receptor and sex steroid hormone receptors. The DNA binding domain binds to two types of response elements: interferon (IFN)-stimulated response element (ISRE) and IFN-γ-activated elements (GAS), the latter being an inverted repeat of TTC/TN3-4A/GAA that allows parallel dimer binding in a symmetric fashion. The SH2 domain is needed for efficient receptor recruitment, parallel dimer orientation, and subsequent DNA binding. Importantly, most somatic GOF mutations of STAT3 and STAT5B occur in hematopoietic cancers within the SH2 domain, further emphasizing its importance for STAT function, particularly in blood cells. In addition to facilitating the transcriptional activity of STATs, the transactivation domain at the C-terminus is also involved in phosphatase docking and subsequent STAT inactivation via removal of phosphates from tyrosine or serine residues. Excessive levels of reactive oxygen species (ROS) can inactivate the catalytic function of phosphatases, further explaining the loss of negative regulation in disease processes [15,16,19].

It is critical that activated JAK-STAT proteins are tightly regulated or silenced when cytokine or growth factor responses are terminated. This is mainly achieved through the induction of negative feedback loops via suppressor of cytokine signaling (SOCS) proteins or tyrosine phosphatases. The latter are also partly under the control of JAK kinases, which provide specificity and selectivity of the signal transduction in a cell type-specific manner [8].

In summary, STAT proteins form homo- or heterodimers and translocate from the cell membrane into the nucleus, which is partly regulated by serine/threonine kinases. Here, they efficiently regulate transcription by binding to promoter and enhancer regions of cytokine-inducible genes, providing growth and survival signals as well as steering metabolism in a transient manner in normal cells, but persistently in cancer cells [9,10,14,26,27].

## 3. O-GlcNAcylation as an Essential Post-Translational Modification

O-GlcNAc is a highly dynamic serine- or threonine-linked monosaccharide modification that marks thousands of proteins. This post-translational modification (PTM) plays a major role in the development of cancer, neurodegeneration, and diabetes. In contrast to the high number of different phosphatases and kinases, the O-GlcNAc modification is regulated by only two enzymes: O-GlcNAc transferase (OGT) as a writer and O-GlcNAcase (OGA) as an eraser. Both are essential genes and their loss is embryonically lethal. They promote the proliferation and survival of different cell types, including neurons, fibroblasts, and embryonic stem cells. A loss of these enzymes disturbs protein glycosylation and, therefore, proper cell development during embryonic development [28,29,30,31]. The donor for O-GlcNAc modification—uridine diphosphate-GlcNAc (UDP-GlcNAc)—is generated in the hexosamine biosynthetic pathway (HBP) [28,32,33,34].

**Figure 2:**
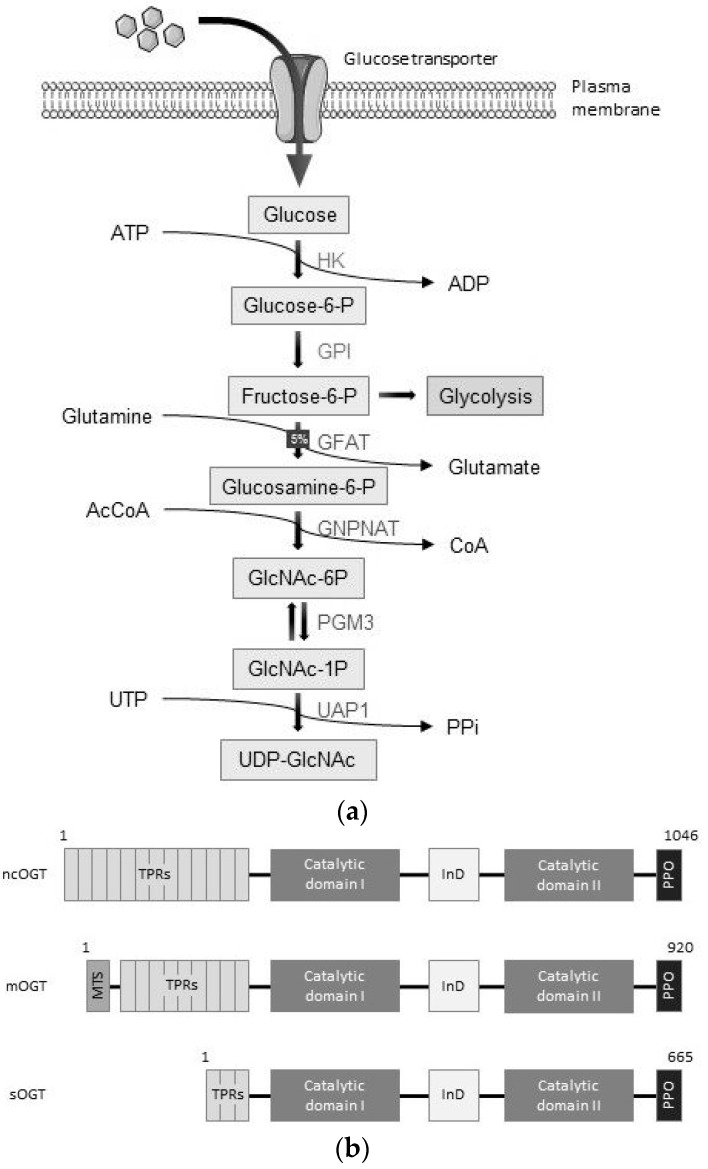

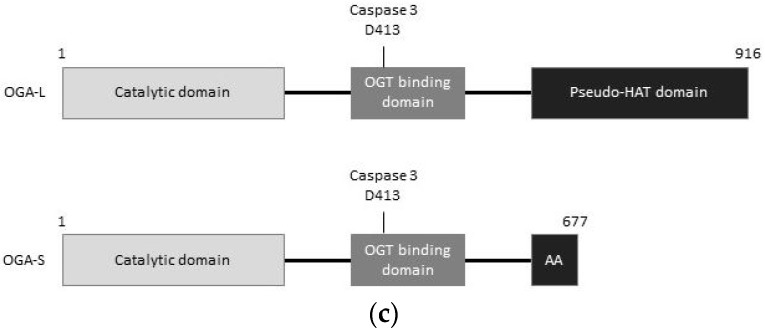
The hexosamine biosynthetic pathway (HBP) and the two key enzymes: O-GlcNAc transferase (OGT) as a writer and O-GlcNAcase (OGA) as an eraser with their isoform structures. (**a**) The donor for O-GlcNAcylation, UDP-GlcNAc is synthesized when glucose enters the HBP. Here, glutamine serves as a nitrogen donor and AcCoA serves as an acetyl donor. More detailed pathway descriptions can be found in the text [27,29,35,36]. (**b**) Three OGT isoforms are known: ncOGT, mOGT, and sOGT. They are generated by alternative splicing and they largely differ in the number of tetratricopeptide repeats (TPRs) at the N-terminus, which is necessary for protein–protein interactions. The catalytic domains I and II are responsible for binding to UDP-GlcNAc and transferring the O-GlcNAc group to serine or threonine residues on target proteins. The OGT enzyme can, for example, be recruited to the cell membrane via the PIP-binding activity domain (PPO) domain as characterized in response to insulin receptor activation by insulin [28,29,37,38]. (**c**) There are two known isoforms of OGA: a long (OGA-L) and a short (OGA-S). Both share a catalytic domain and an OGT binding domain. The long isoform contains a pseudo-HAT domain, which is located at the C-terminus, and the short isoform has only a short amino acid stretch with less known function. Both isoforms have a cleavage site for caspase 3 at position D413, which is associated with apoptosis [28,29,39].

The HBP is a non-canonical pathway in glucose metabolism, in which, depending on the metabolic state, 1–5% of intracellular glucose is processed to UDP-GlcNAc. To generate the donor for O-GlcNAcylation processes, six enzymatic steps are essential, and two of these are shared with the glycolysis pathway. First, cytoplasmic glucose molecules are phosphorylated by hexokinase 1/2 (HK) to generate glucose-6-phosphate. Second, glucose-6-phosphate is then converted into fructose-6-phosphate by phosphoglucose isomerase (GPI). In the third step, glutamine acts as a nitrogen donor to form glucosamine-6-phosphate via the enzyme glutamine: fructose-6-phosphate transaminase (GFAT). Thereafter, N-acetylglucosamine-6-phosphate (GlcNAc-6P) is generated by glucosamine-phosphate N-Acetyltransferase (GNPNAT) using acetyl coenzyme A (AcCoA). Subsequently, the enzyme GlcNAc phosphomutase (PGM3) converts GlcNAc-6P into GlcNAc-1P. In the final step, GlcNAc-1P and uridine triphosphate (UTP) are combined to form UDP-GlcNAc (Figure 2a) [29,35,36].

Although there is no consensus sequence known for O-GlcNAc modification, loop structures or intrinsically disordered (ID) regions are much more likely to be modified than α-helices or β-sheet regions [28,40]. Screening O-GlcNAc sites on peptides defined the preferred modification sequence as (TS)(PT)(VT)**S/T**(RLV)(ASY) (modification site indicated in bold) [41]. Nevertheless, O-GlcNAcylation is not restricted to this motif, and other sites may also be modified.

OGT is the only enzyme known to add O-GlcNAc modification to proteins. There are three known OGT isoforms, generated by alternative splicing. All splice variants are distinguished by the number of tetratricopeptide repeats (TPRs). The longest, nucleocytoplasmic OGT (ncOGT, 110 kDa), is located in both the cytoplasm and nucleus. An alternative start codon in the fourth exon is used for the expression of mitochondrial OGT (mOGT) with a size of 103 kDa. This variant also has an additional mitochondrial targeting site (MTS) and is involved in glycosylation of mitochondrial proteins. Though the existence of mOGT has been reported in human cells, its function is still controversial, especially in other species. The third isoform, short OGT (sOGT), is 70 kDa, is derived from a longer transcript and is localized, along with ncOGT, in the cytoplasm and the nucleus [28,29,37,38,42].

All three OGT variants contain a glycosyltransferase catalytic activity region, divided into the catalytic domain (CD) I, which is the catalytically active site of the protein, and the CD II which binds UDP-GlcNAc. Both are separated by an intervening domain (InD), whose function is not clarified so far (Figure 2b). The TPRs at the N-terminal region of OGT are found in a wide variety of proteins, from bacteria to human, and they facilitate protein–protein interactions. They are involved in many cellular processes, such as cell cycle regulation, transcriptional control, protein folding, and stress responses. In OGT, the TPRs have an essential function in protein recruitment for O-GlcNAcylation [34,41]. OGT is recruited to the plasma membrane via the PIP-binding activity domain (PPO) in response to insulin regulating glucose homeostasis, where it interacts with phosphatidyl-inositol-3-phosphate (PIP3) [28,29,37,38].

Recently, a protein was discovered that is responsible for O-GlcNAcylation of extracellular domains of transmembrane proteins, namely the EGF domain-specific OGT (EOGT). It is localized to the ER and binds, in contrast to conventional OGT, to a conserved binding motif: a serine or threonine between the fifth and the sixth cysteine residue of EGF repeats. Although EOGT and OGT share very few sequence similarities, both are regulated by the HBP flux and have the same function of attaching O-GlcNAc to serine and threonine residues [43,44].

OGA is the enzyme responsible for removal of the O-GlcNAc modification from proteins. It exists in two splice variants: long (OGA-L, 103 kDa) and short (OGA-S, 76 kDa) (Figure 2c). Both variants share a catalytic amidase domain and an OGT binding region. At the position D413, OGA can be cleaved by caspase 3, which is associated with apoptosis. At the C-terminal end, OGA-L contains a pseudo-histone acetyltransferase (HAT) domain, which shares similarities with the domain found in HATs, but has no functional HAT activity. This leads to controversies about the actual function of this domain. In contrast to OGA-L, OGA-S lacks this pseudo-HAT domain, but has a unique amino acid extension, which is necessary to attach to lipid droplets. Therefore, OGA-L is mainly localized to the cytoplasm and the nucleus, whereas OGA-S resides inside of lipid droplets [28,29,39,45].

## 4. O-GlcNAc is Crucial for JAK-STAT Pathway Functions

Proteins can be covalently modified by the addition of different modifications, such as phosphate, methyl, acetyl, or glucose groups, on specific amino acids. These PTMs influence the biochemical properties of a protein or its function. Protein phosphorylation can also be used to transmit a signal or to inactivate proteins. Many other PTMs are known, such as ubiquitination, sumoylation, neddylation, or succinylation, which can alter protein function or mark them for degradation [46,47,48,49,50,51].

Target gene spectra of STAT proteins are shaped by cell type-specific interactions and PTMs, splicing of STAT transcription factors, or proteolytic processing, where disturbances of these processes contribute to disease mechanisms. The most crucial PTM in the JAK-STAT pathway is tyrosine phosphorylation, which orchestrates these proteins to become active. Notably, other PTMs can influence STAT5 activity. Sumoylation and methylation inhibit the function of STAT5, whereas glycosylation via O-GlcNAc is required for STAT5 activity, promoting increased pYSTAT5 levels and increased transcriptional capacity [7,52,53]. Therefore, various PTMs should be considered when studying abnormal activities of STAT5 proteins in disease processes such as hematopoietic cancers.

In cases where the PTM balance is altered, aberrant protein function will likely result. For example, the W515L mutation in the TPO receptor (TPOR) leads to hyperphosphorylation of JAK2, STAT3, STAT5, and other important signal transducers, promoting myeloid cell transformation. This *TPOR* mutation is the most frequent mutation in JAK2_V617F_-negative essential thrombocytopenia or myelofibrosis [54].

It was shown that oxidative stress induced by hypoxia shifts glycolysis to the HBP. It is interesting that STAT5 can directly regulate hypoxia inducible factor (HIF)1β, whereas STAT3 directly controls HIF1α. Consequently, both STAT3 and STAT5 are involved in angiogenesis, and they can regulate metabolic processes under hypoxic conditions [55]. Surprisingly, different metabolic conditions do not correlate with protein O-GlcNAcylation status in general. Some studies show that glucose starvation causes higher protein O-GlcNAcylation, likely caused by secondary effects that have not been fully elucidated [28,56,57]. Furthermore, it was shown that a high O-GlcNAc status can increase cancer cell resistance against chemotherapeutic drugs, such as doxorubicin [58]. Therefore, O-GlcNAc is a highly interesting and versatile PTM that could be an attractive target for new anti-cancer drug development, particularly in the context of oncogenic STAT5A in hematopoietic cancers.

As previously mentioned, O-GlcNAc modification influences protein functionality and activity. Based on wheat germ agglutinin (WGA) affinity chromatography, STAT1/3/5A/5B/6 are glucose-modified on a threonine or serine residue [59]. So far, further detailed mapping was only performed for STAT5A and STAT5B. Here, the glucose was shown to be attached to T92 within an ATQL tetrapeptide motif in an N-domain α-helix that was conserved in both STAT5 gene products (Figure 1b) [7]. Blocking O-GlcNAcylation by mutation of T92 to alanine substantially decreased tyrosine phosphorylation of oncogenic STAT5A. Given that STAT5A tyrosine phosphorylation is important for its interaction with CBP/p300 HATs, this may suggest that blocking O-GlcNAcylation at T92 might also reduce this interaction and subsequently influence chromatin accessibility [7,28,60].

In the case of STAT5B, which is more frequently mutated in cancer, the influence of O-GlcNAc on phosphorylation could not be demonstrated, even in the presence of the strongly activating STAT5B N642H mutation [7,59]. These findings might suggest that O-GlcNAcylation of hyperactive STAT5A or STAT5B variants regulates distinct functions. Indeed, STAT5A appears to be less oncogenic than STAT5B, at least in certain models [61], and since both proteins can make homo- or heterodimers and have similar gene transcription profiles, such distinct regulation of STAT5A and STAT5B by O-GlcNAcylation could potentially facilitate this oncogenic specificity. Notably, serine-phosphorylated mitochondrial STAT3 is essential for RAS-driven transformation. Currently, it has not been investigated if STAT3 is also O-GlcNAcylated and if it interacts with OGT [62]. It is tempting to speculate that such a scenario may occur, but this still needs to be tested experimentally.

If the site of O-GlcNAcylation is in close proximity to a phosphorylation site, both can sterically compete with each other. Therefore, this suggests that a change in protein function and stability can depend on the antagonism of a phosphorylation or glycosylation modification at a given threonine or serine residue. Thus, O-GlcNAcylation can influence other PTMs, such as phosphorylation of proteins [7,28,63]. There are also additional PTMs, such as sumoylation, ubiquitination, or acetylation, known to modify STAT5 [48]. Therefore, it cannot be excluded that O-GlcNAcylation may also influence these PTMs, or vice versa. Furthermore, phosphorylation positively regulates the activity and stability of OGT [64,65], and a number of ubiquitin modifications have also been detected on the OGT protein by mass spectrometry [66], although their function in regulating the protein has not been experimentally determined. Therefore, PTMs also play an important role in regulating the O-GlcNAcylation pathway.

**Figure 3:**
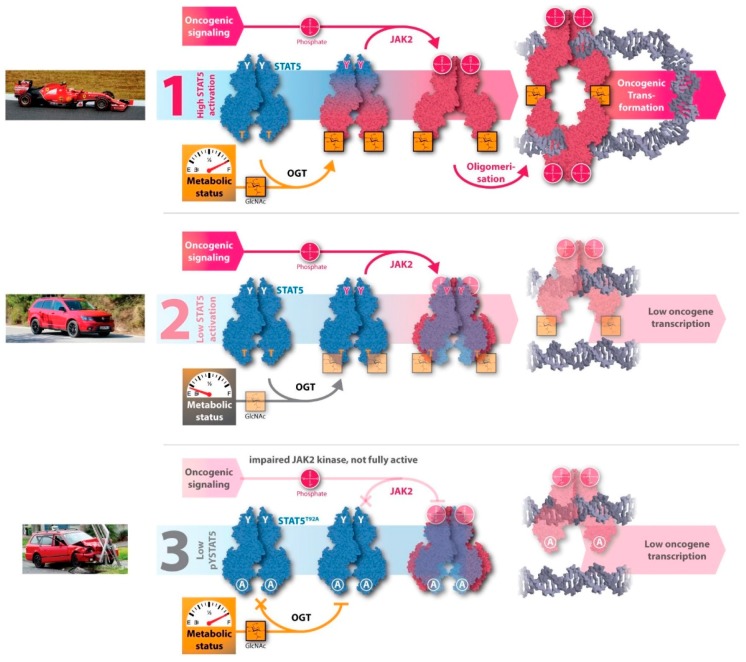
Inhibition of O-GlcNAcylation of oncogenic STAT5A. Three different modes of action explain oncogenic (1) or normal (2,3) function of STAT5A. A racecar, a family car, or a damaged car serve as a metaphor to illustrate oncogenic transcription processes. In the case of gain-of-function (GOF) mutated STAT5A and sufficient nutrient supply, then increased ‘power’ and a full ‘fuel tank’ allows oncogenic transformation to occur (1). In the case of low metabolic status, and therefore less ‘power’ (2), the cancer cells driven by oncogenic STAT5A cannot divide as they lack the ‘fuel’ required for important cellular processes. Independently from the metabolic status, blocking O-GlcNAcylation by mutation, illustrated by a damaged car, also lowers oncogenic transcription in the context of a GOF oncogenic variant of STAT5A (3). When the critical O-GlcNAcylation site is abolished by mutation, then transformation is lost, and cancer cells driven by oncogenic STAT5A revert to normal cell signaling [7].

Mutations, such as STAT5A_S710F_, increase its tyrosine phosphorylation status and thereby the activity and gene transcription capacity. Consequently, control over cell proliferation decreases and cancer can emerge and develop. Inhibiting O-GlcNAcylation of the hyperactive STAT5A variant normalizes oncogenic transcription of target genes back to wild type level. There are several indirect approaches to block O-GlcNAcylation of STAT5A. First, glucose depletion can reduce the flux into the HBP and thereby lower the UDP-GlcNAc concentration, resulting in decreased O-GlcNAcylation. This concept is consistent with the idea that a low metabolic status causes a low glycosylation rate (Figure 3). Second, blocking the generation of UDP-GlcNAc by small molecular weight chemical inhibitors, such as alloxan or 6-diazo-5-oxo-L-norleucine (DON) that block or reduce the HBP flux, will cause a reduction in UDP-GlcNAc synthesis. DON does not directly inhibit OGT, but it inhibits GFAT in the HBP (Figure 3) [7].

Third, the genetic approach of mutating T92 to alanine removes the target substrate motif and thereby inhibits O-GlcNAcylation of STAT5A. Bone marrow transplantation into irradiated mice demonstrated that the T92A mutation, and the subsequently abolished O-GlcNAcylation, prevented oncogenic transformation driven by constitutively active STAT5A, yet still resulted in overall stable hematopoiesis and normalization of proto-oncogene transcription of c-myc, D-type cyclins, OSM, or Bcl-2 family members.

In many cancers, especially in most hematologic cancers, STAT5 activation and its oncogenic gene expression is not only enhanced, but also kept persistent, whereas signaling involving activation of STAT5 is rather transient under physiological conditions. Cancer-specific metabolic changes enhance glycosylation, which subsequently modulates STAT5 activity through enhanced tyrosine phosphorylation. Reducing O-GlcNAc by changing the metabolic status, via glucose depletion or hypoxia, can reduce oncogenic transcription of STAT5 target genes to wild type levels, as was shown in the context of the GOF mutation of STAT5A [7]. Here, it should be mentioned that it is possible that these stress conditions may also increase O-GlcNAcylation of some proteins by increased OGT expression caused by a reduced HBP flux [57].

Our findings on the control of O-GlcNAc-modified STAT5A can also have consequences for many hematopoietic cell types that have distinct responses to low or high pYSTAT5 levels, giving rise to distinct T-cell effector or Treg functions or subsets, different B-cell (B1 versus B2) subsets, or myeloid cell functions (M1 to M3 macrophages) [67]. The discussed work and mechanism of STAT5 O-GlcNAcylation raises the possibility of developing new anti-cancer therapy strategies by inhibiting O-GlcNAcylation of hyperactive STAT5 in combination with inhibition of hyperactive tyrosine kinase signaling. In addition, we have also detected O-GlcNAcylation of JAK kinases, and our labs are actively engaged in mapping and exploring the mechanisms into the regulatory functions of this modification in essential players of the JAK-STAT core cancer pathway. We hypothesize that O-GlcNAcylation of JAK kinases may regulate the O-GlcNAcylation and/or activation of STATs.

In summary, metabolic and transcriptional control through O-GlcNAcylation of key proteins of the JAK-STAT pathway has therapeutic targeting potential, but there is clearly still more to investigate in order to understand the full consequences of this modification in cancer.

## Abbreviations

AcCoA	Acetyl Coenzyme A
AML	Acute Myeloid Leukemia
CALR	Calreticulin
CD	Catalytic domain
CML	Chronic Myeloid Leukemia
DON	6-diazo-5-oxo-L-norleucine
EOGT	EGF domain-specific OGT
EPO	Erythropoietin
GFAT	Glutamine Fructose-6-phosphate Transaminase
GM-CSF	Granulocyte-Macrophage Colony-Stimulating Factor
GNPNAT	Glucosamine-Phosphate N-Acetyltransferase
GOF	Gain Of Function
GPI	Phosphoglucose Isomerase
HAT	Histone Acetyl Transferase
HBP	Hexosamine Biosynthetic Pathway
HIF	Hypoxia Inducible Factor
HK	Hexokinase
ID	Intrinsically disordered
IFN	Interferon
IL	Interleukin
InD	Intervening domain
JAK	Janus kinase
MCP	Monocarboxylate transporter
MPN	Myeloproliferative neoplasm
MTS	Mitochondrial targeting site
OGA	O-GlcNAcase
O-GlcNAc	O-linked β-N-acetyl glucosamine
OGT	O-GlcNAc transferase
OSM	Oncostatin M
PDHX	Pyruvate dehydrogenase protein X component
PGM3	GlcNAc Phosphomutase
PIP3	Phosphatidyl-inositol-3-phosphate
PPO	PIP-binding activity domain
PTM	Post-translational modification
ROS	Reactive oxygen species
SH	Src homology
SOCS	Suppressor of cytokine signaling
STAT	Signal transducer and activator of transcription
TCA	Tricarboxylic acid
TPO	Thrombopoietin
TPOR	Thrombopoietin receptor
TPR	Tetratricopeptide repeat
TYK	Tyrosine kinase
UDP-GlcNAc	Uridine diphosphate-GlcNAc
UTP	Uridine triphosphate
WGA	Wheat germ agglutinin
